# Gut Microbiome Composition is Associated with Age and Memory Performance in Pet Dogs

**DOI:** 10.3390/ani10091488

**Published:** 2020-08-24

**Authors:** Eniko Kubinyi, Soufiane Bel Rhali, Sára Sándor, Attila Szabó, Tamás Felföldi

**Affiliations:** 1 Department of Ethology, ELTE Eötvös Loránd University, 1117 Budapest, Hungary; belghalisoufiane@gmail.com (S.B.R.); sandorsara@gmail.com (S.S.); 2 Department of Microbiology, ELTE Eötvös Loránd University, 1117 Budapest, Hungary; attila.szabo.ttk@gmail.com (A.S.); tamas.felfoldi@gmail.com (T.F.)

**Keywords:** gut microbiome, dog, cognition, aging

## Abstract

**Simple Summary:**

The intestinal tract affects the brain through metabolites produced by gut-inhabiting bacteria. In this study, we show that the number of errors the dogs commit in a short-term memory test and also their age is linked to the gut microbiome composition. The proportion of Fusobacteria is lower in older animals. Dogs with better memory performance (i.e., fewer mistakes) have relatively fewer Actinobacteria in their fecal samples collected right after the behavior test. This result is in agreement with the high abundance of some Actinobacteria in the gastrointestinal tract of persons living with Alzheimer’s disease. Links between memory performance and gut microbiota have been reported on rodents but not on dogs before. The research opens up new venues in canine aging and neurodevelopmental research.

**Abstract:**

Gut microbiota can crucially influence behavior and neurodevelopment. Dogs show unique similarities to humans in their physiology and may naturally develop dementia-like cognitive decline. We assessed 29 pet dogs’ cognitive performance in a memory test and analyzed the bacterial 16S rRNA gene from fecal samples collected right after the behavioral tests. The major phyla identified in the dog microbiomes were Bacteroidetes, Firmicutes, and Fusobacteria, each represented by >20% of the total bacterial community. Fewer Fusobacteria were found in older dogs and better memory performance was associated with a lower proportion of Actinobacteria. Our preliminary findings support the existence of links between gut microbiota, age, and cognitive performance in pet dogs.

## 1. Introduction

The intestinal tract harbors the most abundant and diverse collection of microbes in the body. Several studies performed in rodents suggest that neurodevelopment is affected by gut microbiota [1,2,3]. In humans, intestinal microbiome composition has been linked to psychiatric conditions, such as depression, anxiety, and autism, as well as neurodegenerative disorders, including Parkinson’s and Alzheimer’s disease [4]. The primary path through which the gut microbiome is thought to affect behavior are metabolites produced by gut-inhabiting bacteria. Some of them (such as *Lactobacillus* and *Bifidobacterium*) can produce neurotransmitters or other molecules, including γ-aminobutyric acid (GABA), amino-acid derivatives (e.g., serotonin, melatonin, and histamine), fatty-acid derivatives (e.g., acetylcholine), or catecholamines (e.g., dopamine and norepinephrine [5,6]). It has therefore been postulated that a regulatory pathway, called the gut–brain axis, exists between the enteric nervous system of the gastrointestinal tract (GIT) and the central nervous system [7]. 

Dogs have become a valuable model of human complex traits and disorders [8,9]. The wide range of expected lifespans, a natural risk to develop dementia, and an environment shared with humans, has also made dogs a promising model organism in aging research (for a review see [10]). Since laboratory dogs represent a limited sample of the natural genetic and environmental variability found in human populations and companion (or pet) dogs, the latter has gained more popularity in aging research recently [11,12,13,14,15,16,17,18]. Their behavior also differs from that of laboratory dogs [19] in ways that make the companion dog a more ecologically valid model of human aging.

The gut microbiome of dogs is more similar to that of humans than that of mice and pigs [20]. Although studies on dogs’ microbiota and their connection to disease prevalence and aging are still scarce [21,22], they suggest that nutritional supplements may improve some of the symptoms of age-related pathological cognitive decline in dogs [23].

Next-generation DNA sequencing techniques have enabled the detailed determination of the taxonomic composition and also the potential functions of the microorganisms, gaining a better understanding of microbial–host interactions [24]. We selected this technique for the analysis of the gut microbiome of a group of companion dogs and examined possible links with age and cognitive performance. Based on a study in mice, we predicted that memory performance is linked to *Lactobacillus* [25] and that age would be associated with less diverse microbiota [21,26] and/or less abundant lactobacilli [24].

## 2. Materials and Methods

### 2.1. Ethics Statement

The behavioral observations conducted in this study complied with national (Hungarian law (‘1998. évi XXVIII. Törvény’ 3. §/9.—The Animal Protection Act)) and EU legislation, as well as institutional guidelines. The Hungarian “Animal Experiments Scientific and Ethical Committee” approved the experimental procedures under the numbers: PE/EA/2019-5/2017. The owners provided written consent for their dogs’ participation. The information included the owners’ right to withdraw their consent at any time. The owners could at any point decline to participate with their dog and could request their data not to be used and/or deleted after collection. Non-invasive behavior tests are not considered as an animal experiment and are therefore allowed to be conducted without any special permission from the University Institutional Animal Care and Use Committee (UIACUC). The study was performed in strict accordance with the recommendations in the International Society for Applied Ethology guidelines for the use of animals in research. 

### 2.2. Subjects

Companion dogs (N = 29, 14 males, age range: 3 to 13-year-old, mean age (SD): 9.7 (2.6) years, mean weight (SD): 23.3 (14.3) kg; 1 Bernese mountain dog, 6 Border collies, 2 Cairn terriers, 1 German wirehaired pointer, 1 Labrador retriever, 12 Mixed breeds, 1 Moscow watchdog, 2 Vizslas, 3 Whippets) living in Hungarian households were included in the study. The dogs participated in a larger study investigating the behavioral signs of aging [27]. These dogs had defecated right after the behavior tests and were thus included here due to the availability of fecal samples. Based on the owners’ reports, dry food was the main diet for 8 dogs, cooked food for 1 dog, raw meat for 2, and mixed food (i.e., no “main” food type could be specified) for 18 dogs. Three dogs received (unspecified) vitamins/nutritional supplements daily, 7 often, 6 rarely, 13 never. Three dogs were classified by their owners as “obese”, 1 as “thin” and 25 as “normal” weight. In the case of 6 individuals, fecal samples could also be collected after a 3-month interval, since they again participated in the behavior test and defecated.

### 2.3. 16S rRNA Gene Amplicon Sequencing

Fecal samples were collected immediately after the dogs had participated in the behavior tests (see below). The samples were collected after spontaneous defecation and conserved at −80 °C within 15 min. The gut microbiome analysis was performed on the samples using amplicon sequencing of the bacterial 16S rRNA gene. Total genomic DNA extraction was performed using the QIAamp Power Fecal DNA kit (Qiagen, Venlo, Netherlands) following the instructions given by the manufacturer. DNA sequencing was performed on an Illumina MiSeq platform using MiSeq standard v2 chemistry as a service provided by the Genomics Core Facility RTSF, Michigan State University, East Lansing MI, USA. Methodological details and instructions for the bioinformatic analysis are given in [28]. Briefly, sequences were processed using the Mothur software [29] reads were quality-filtered, chimeras (identified by the program described in [30]), and singletons were removed from the dataset. Sequences were preclustered and operational taxonomic units (OTUs) were defined at a 97% nucleotide sequence similarity level. Taxonomic assignment applying 1000 iterations and bootstrap with a cutoff at 80 was carried out using the ARB SILVA SSU Ref NR 132 [31] database. Reads of non-bacterial origin were removed from the dataset. Raw amplicon sequence data were submitted to NCBI under BioProject ID PRJNA603883.

### 2.4. Assessing the Cognitive Performance: Memory Test

The detailed procedure of the behavior test is described in [27,32]. In short, five identical, open containers were positioned in equal distance from each other in a semi-circular arrangement, each container being two meters away from the starting position of the dog. A dog witnessed the baiting of one of the containers with a treat and then left the room for 30 s. Then the dog was returned to the starting position and was set free to find the baited container. The task was repeated five times and each container was baited once in a semi-randomized order. All dogs obtained the reward within 30 s in each trial. The measured variable was “memory test mistakes”: the number of investigating an unbaited container during the five trials (since the dogs could visit any number of containers until they found the right location). 

### 2.5. Statistical Analysis and Ordination

For the statistical analysis of amplicon sequencing data, the subsampling of reads was performed to the read number of the smallest dataset (N = 28,489). The test–retest reliability of gut microbiota composition was investigated with Wilcoxon signed-rank tests at the phylum-level, which did not detect a change in the microbiota composition. We carried out four regression tree analyses, one for age and one for memory mistakes on both the phylum level and genus level, to examine their relationship with the bacterial phyla, richness estimators (sobs, ACE, Chao-1), and diversity values (inverse Simpson) of the gut microbiota. We chose regression trees because they are ideal for analyzing complex numeric and/or categorical data and detecting non-linear relationships (see description in [33]). We used the chi-square automatic interaction detection (CHAID) method and specified the minimum number of cases as 29 for parent nodes and 9 for child nodes. IBM SPSS v25 and non-metric multidimensional scaling (NMDS) ordination with R (R Core Team, 2017) were used for the analyses.

## 3. Results

### 3.1. Gut Microbiome Composition

A total of 1,857,465 high-quality bacterial 16S rRNA gene sequences were obtained from the first fecal samples (51,596 ± 14,774 reads per sample). Good’s coverage values were higher than 0.99 in all cases, which indicated that sequencing depth was sufficient to recover all major taxa. The average length of sequences was 452 nt, which allowed for genus-level taxon identification. 

**Figure 1:**
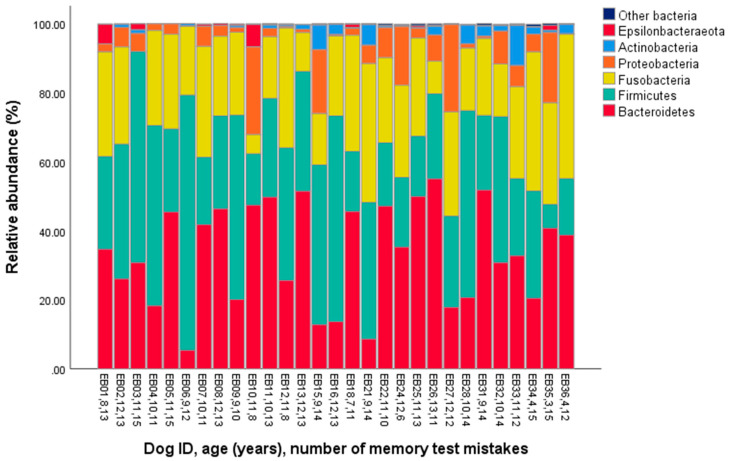
Phylum-level gut microbiota composition in the fecal samples of 29 companion dogs.

**Figure 2:**
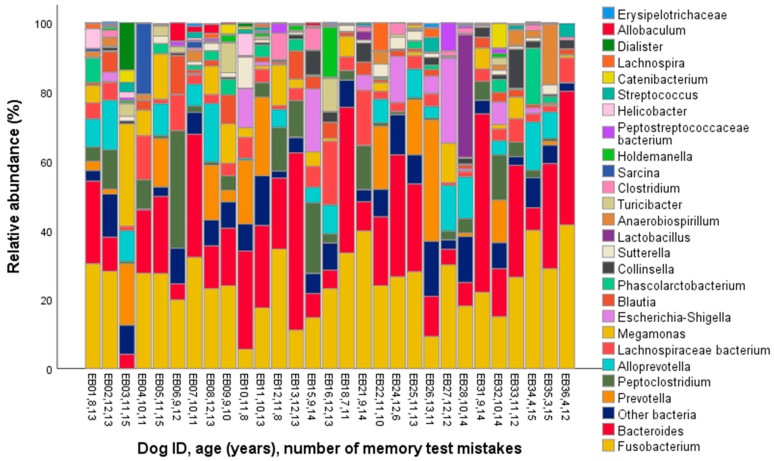
Genus-level gut microbiota composition in the fecal samples of 29 companion dogs.

The major phyla identified in the dog microbiomes were Bacteroidetes, Firmicutes, and Fusobacteria (represented by >20% of the total bacterial community on average, Table 1, Figure 1), and the main genera were *Fusobacterium*, *Bacteroides*, *Prevotella*, *Peptoclostridium,* and *Alloprevotella* (>5% on average, Table 1, Figure 2).

### 3.2. Gut Microbiota Composition Associations with Age and Memory Performance

**Figure 3:**
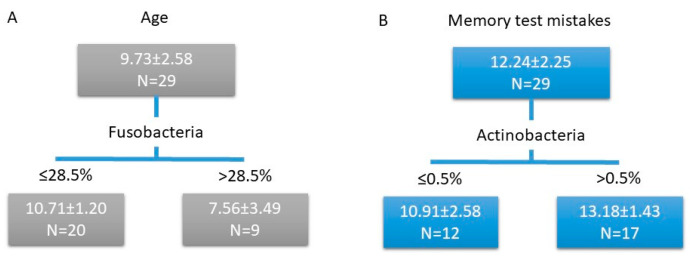
Regression tree model for age (**A**) and memory test mistakes (**B**). Scores in the rectangles (subgroups): mean ± SD.

Higher age was associated with a lower proportion of Fusobacteria (F = 13.349, *p =* 0.010, Figure 3A). Higher cognitive performance (i.e., fewer errors in the short-term memory-test) was linked to a lower proportion of Actinobacteria (F = 9.203, *p =* 0.042, Figure 3B). According to NMDS ordination, the general composition of bacterial communities was not directly linked with cognitive performance (Figure S1). No other significant correlations were revealed for other taxa (at the phylum level or genus level) or diversity and richness measures.

## 4. Discussion

In this study, we measured pet dogs’ microbiome composition and examined its links with age and memory performance. The most abundant phyla were Bacteroidetes and Firmicutes (33% both), next the Fusobacteria (24%), followed by Proteobacteria (7%) and Actinobacteria (2%). These proportions are comparable to the results of [34]. They described the following fecal microbiome composition in six healthy dogs: Bacteroidetes (31–34%), Firmicutes (14–28%), Fusobacteria (23–40%), Proteobacteria (~7%), and Actinobacteria (1%). Other studies identified the same predominant phyla, but the proportions varied greatly within and between the studies (e.g., [35,36,37]). Differences likely arise due to factors such as breed, age, diet, and living conditions [38]. Based on a sub-sample, the microbiome composition does not seem to have changed notably within a 3-month interval. Other works [37,39] also indicate that a dog’s microbiome composition may remain relatively constant for several weeks or months, even when moderate changes in the diet are applied.

Regarding age-related differences, we could not confirm the results of previous studies performed on purebred dogs. Common findings are the decrease in gut microbiome diversity and an abundance of lactobacilli with increased age [21,24,26]. In our sample, encompassing several breeds, we instead observed a significant decline in Fusobacteria with age, not described previously in the literature. In contrast to our result, Fusobacteria showed not lower but higher prevalence (the percentage of positive samples) in human centenarians than in elderly and young adults [40]. In agreement with the possibly inverse role of Fusobacteria in the aging of humans and dogs, a higher proportion of Fusobacteria is associated with a healthy microbiota [17,22,41] (and obesity [42]) in dogs, while in humans they are implicated in dysbiosis, e.g., in inflammatory bowel disease [42], colorectal cancer [42], and acute appendicitis [43]. Fusobacteria are more abundant in carnivores than in humans and our data support the assumption of other studies [44,45,46] that they play a substantially different, possibly beneficial role in the carnivore digestive system. Fusobacteria are also present in a greater relative abundance in the fecal samples of non-aggressive dogs [47] and dogs with access to the outdoors [22,48]. 

Memory performance was also associated with microbiota composition: dogs with lower performance (i.e., more memory test mistakes) had relatively more Actinobacteria in their fecal samples. This result is in agreement with the high abundance of some Actinobacteria in the GIT of Alzheimer’s patients [49]. 

The limitations of the study include a relatively low number of young dogs, a small sample size for testing behavioral associations, and a high variation in breed and feeding regime. This study was observational using a cross-sectional design and our findings must be further validated. On the other hand, the number of dog microbiome samples was comparable to that of recent studies with 30–45 samples (e.g., [21,36,47]). 

## 5. Conclusions

A link between cognitive performance, age, and gut microbiome composition in companion dogs was hypothesized but not described before. Our research, although preliminary regarding the association between cognition and gut microbiome composition, opens up new venues in canine aging and neurodevelopmental research. Dogs have recently also been proposed as natural models of human autism [50], in which the relevance of microbial dysbiosis has been documented [51]. However, the inverse role of Fusobacteria in human and dog dysbiosis and age highlights the need for more studies to investigate the function these bacteria play in the carnivore digestive system.

## Figures and Tables

**Table 1:** Microbial community of companion dogs’ microbiome at the phylum level and genus level in percentages.

**Phylum**	**Mean**	**Standard Deviation**	**Minimum**	**Maximum**
Actinobacteria	1.9	2.7	0.0	11.7
Bacteroidetes	33.3	14.7	5.4	55.1
Epsilonbacteraeota	0.7	1.5	0.0	6.4
Firmicutes	33.0	16.5	6.8	73.9
Fusobacteria	24.4	10.1	0.0	41.9
Proteobacteria	6.5	7.5	0.1	25.4
Other bacteria	0.2	0.2	0.0	0.7
**Genus**	**Mean**	**Standard Deviation**	**Minimum**	**Maximum**
*Allobaculum*	0.5	1.1	0.0	5.4
*Alloprevotella*	5.2	5.3	0.0	16.9
*Anaerobiospirillum*	1.3	3.4	0.0	17.5
*Bacteroides*	21.1	14.1	4.1	51.6
*Blautia*	2.5	2.8	0.0	11.2
*Catenibacterium*	0.6	1.5	0.0	7.1
*Clostridium*	1.0	1.8	0.0	7.6
*Collinsella*	1.6	2.5	0.0	11.3
*Dialister*	0.5	2.6	0.0	13.8
Erysipelotrichaceae bacterium	0.1	0.3	0.0	1.2
*Escherichia-Shigella*	3.1	6.1	0.0	24.5
*Fusobacterium*	24.2	10.0	0.0	41.6
*Helicobacter*	0.6	1.6	0.0	6.4
*Holdemanella*	0.8	2.7	0.0	14.5
*Lachnospira*	0.5	1.5	0.0	8.0
Lachnospiraceae bacterium	5	4.4	0.8	18.5
*Lactobacillus*	1.3	6.6	0.0	35.5
*Megamonas*	4.6	6.3	0.0	29.6
*Peptoclostridium*	5.8	7.5	0.0	34.0
Peptostreptococcaceae bacterium	0.7	1.6	0.0	8.2
*Phascolarctobacterium*	1.8	3.3	0.0	16.3
Prevotella	6.2	9.4	0.0	35.1
*Sarcina*	0.8	3.8	0.0	20.5
*Streptococcus*	0.6	1.1	0.0	3.9
*Sutterella*	1.4	1.8	0.0	9.0
*Turicibacter*	1.1	2.4	0.0	9.5
Other bacteria	7.0	4.0	0.2	15.9

## Supplementary Materials

The following is available online at https://www.mdpi.com/2076-2615/10/9/1488/s1, Figure S1: Two-dimensional non-metric multidimensional scaling (NMDS) plot of the microbial community composition (on genus-level) in the fecal samples of 29 pet dogs (EBs). The plot is based on the Bray-Curtis distance of the bacterial OTUs (OTUs contributed to 70% variance between samples by SIMPER analysis are shown in gray). Color coding of samples is according to the number of mistakes in the short-term memory test.
