# Supplementary material for: Gut Microbiome Composition is Associated with Age and Memory Performance in Pet Dogs
Source: Animals (Basel). 2020 Aug 24;10(9):1488. doi: 10.3390/ani10091488 (PMC7552338; doi:10.3390/ani10091488)
Supplement: Supplementary file 1 [file animals-10-01488-s001.pdf]

animals

MDPI

Supplemental Material

Gut Microbiome Composition Is Associated with Age and Memory Performance in Pet Dogs

Eniko Kubinyi [1,*,†], Soufiane Bel Rhali [1,2,†], Sára Sándor [1], Attila Szabó [2] and Tamás Felföldi [2]

[1] Department of Ethology, ELTE Eötvös Loránd University, 1117 Budapest, Hungary; belghalisoufiane@gmail.com (S.B.R.); sandorsara@gmail.com (S.S.)
[2] Department of Microbiology, ELTE Eötvös Loránd University, 1117 Budapest, Hungary; attila.szabo.ttk@gmail.com (A.S.); tamas.felfoldi@gmail.com (T.F.)
* Correspondence: eniko.kubinyi@ttk.elte.hu
† These authors contributed equally to this work.

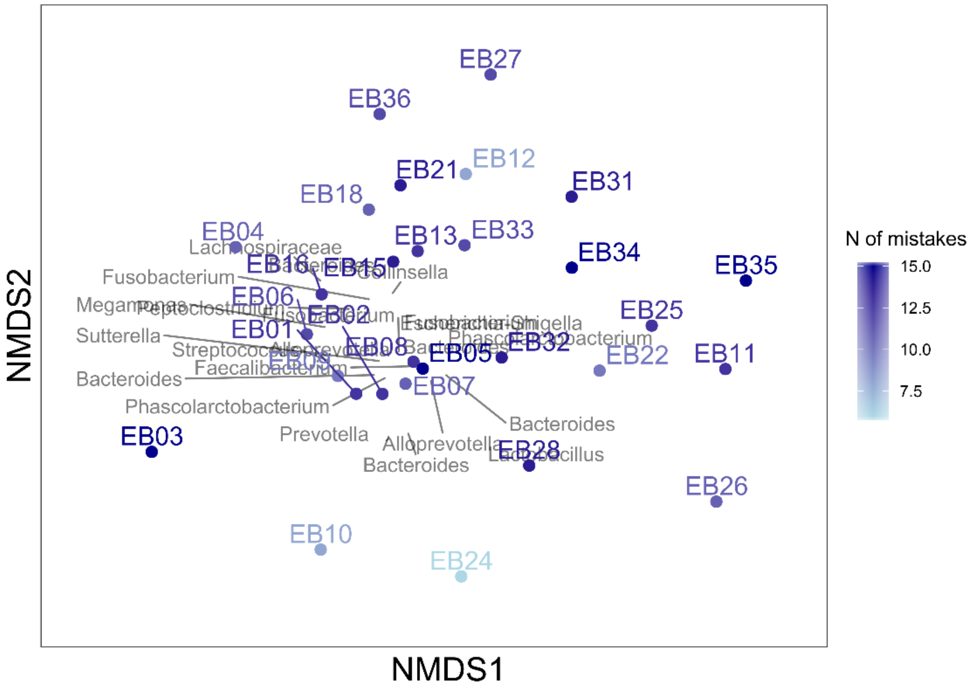

Figure S1. Two-dimensional non-metric multidimensional scaling (NMDS) plot of the microbial community composition (on genus-level) in the fecal samples of 29 pet dogs (EBs). The plot is based on the Bray-Curtis distance of the bacterial OTUs (OTUs contributed to 70% variance between samples by SIMPER analysis are shown in gray). Color coding of samples is according to the number of mistakes in the short-term memory test.
